# Home hemodialysis treatment and outcomes: retrospective analysis of the Knowledge to Improve Home Dialysis Network in Europe (KIHDNEy) cohort

**DOI:** 10.1186/s12882-018-1059-2

**Published:** 2018-10-11

**Authors:** Shashidhar Cherukuri, Maria Bajo, Giacomo Colussi, Roberto Corciulo, Hafedh Fessi, Maxence Ficheux, Maria Slon, Eric Weinhandl, Natalie Borman

**Affiliations:** 1 Royal Wolverhampton Hospital, Renal Services, Wolverhampton, England; 2 Hospital Universitario La Paz, Servicio de Nefrologia, Madrid, Spain; 3 Niguarda Hospital, Nefrologia – Centro Trapianti Rene, Milan, Italy; 4 Policlinic University, Azienda Ospedaliero-Universitaria Consorziale Policlinico, Bari, Italy; 5 Hôpital Tenon, Service de Néphrologie et Dialyses, Paris, France; 6 CHR Clémenceau, Service Néphrologie-Hémodialyse-Transplantation, Caen, France; 7 Hospital de Navarra, Servicio de Nefrologia, Pamplona, Spain; 8 NxStage Medical, Inc., 350 Merrimack Street, Lawrence, MA 01843 USA; 9 Department of Pharmaceutical Care and Health Systems, University of Minnesota, Minneapolis, MN USA; 10 Queen Alexandra Hospital, Wessex Kidney Centre, Portsmouth, England

**Keywords:** Adequacy, Antihypertensive medication, Home hemodialysis, Intensive hemodialysis, Kidney transplant, Lactate, Low-flow dialysate, Ultrapure dialysate

## Abstract

**Background:**

Utilization of home hemodialysis (HHD) is low in Europe. The Knowledge to Improve Home Dialysis Network in Europe (KIHDNEy) is a multi-center study of HHD patients who have used a transportable hemodialysis machine that employs a low volume of lactate-buffered, ultrapure dialysate per session. In this retrospective cohort analysis, we describe patient factors, HHD prescription factors, and biochemistry and medication use during the first 6 months of HHD and rates of clinical outcomes thereafter.

**Methods:**

Using a standardized digital form, we recorded data from 7 centers in 4 Western European countries. We retained patients who completed ≥6 months of HHD. We summarized patient and HHD prescription factors with descriptive statistics and used mixed modeling to assess trends in biochemistry and medication use. We also estimated long-term rates of kidney transplant and death.

**Results:**

We identified 129 HHD patients; 104 (81%) were followed for ≥6 months. Mean age was 49 years and 66% were male. Over 70% of patients were prescribed 6 sessions per week, and the mean treatment duration was 15.0 h per week. Median HHD training duration was 2.5 weeks. Mean standard *Kt/V*_urea_ was nearly 2.7 at months 3 and 6. Pre-dialysis biochemistry was generally stable. Between baseline and month 6, mean serum bicarbonate increased from 23.1 to 24.1 mmol/L (*P* = 0.01), mean serum albumin increased from 36.8 to 37.8 g/L (*P* = 0.03), mean serum C-reactive protein increased from 7.3 to 12.4 mg/L (*P* = 0.05), and mean serum potassium decreased from 4.80 to 4.59 mmol/L (*P* = 0.01). Regarding medication use, the mean number of antihypertensive medications fell from 1.46 agents per day at HHD initiation to 1.01 agents per day at 6 months (*P* < 0.001), but phosphate binder use and erythropoiesis-stimulating agent dose were stable. Long-term rates of kidney transplant and death were 15.3 and 5.4 events per 100 patient-years, respectively.

**Conclusions:**

Intensive HHD with low-flow dialysate delivers adequate urea clearance and good biochemical outcomes in Western European patients. Intensive HHD coincided with a large decrease in antihypertensive medication use. With relatively rapid training, HHD should be considered in more patients.

## Background

The prevalence of chronic kidney disease (CKD) is growing internationally, partly due to the steadily growing prevalence of diabetes. This has led to a rise in need for renal replacement therapy (RRT); the number of patients receiving RRT is expected to double by 2030 [1]. Between 2013 and 2014 alone, the number of adult patients receiving RRT in the United Kingdom rose 4.0% [2]. Although use of hemodialysis outpaces use of peritoneal dialysis in the UK and almost every other country [3], patients on home hemodialysis (HHD) represent only slightly more than 4% of dialysis patients in the UK [2]. Across all of Europe, patients on HHD represent < 2% of dialysis patients [4]. Compared to in-center hemodialysis, HHD is associated with lower risk of cardiovascular death and hospitalization [5, 6]. This association may reflect increased treatment frequency, which has been shown in randomized clinical trials to reduce left ventricular mass, improve blood pressure control, and lower serum phosphorus [7–12]. HHD also permits patients the flexibility to dialyze at times that they choose. On the other hand, HHD is associated with higher risk of infection-related hospitalization, compared to in-center hemodialysis [6]. Increased treatment frequency may increase risk of vascular access complications [13].

Despite the potential benefits of HHD, utilization of the modality is low. Historically, this has been attributed to cost and logistics. In the UK, funding for HHD setup costs are a challenge for most hospitals, as the therapy is not included in the tariff set by the specialist commissioners. The recommendation from the National Institute of Clinical Excellence (NICE) to provide HHD to all suitable patients has encouraged renal units to improve HHD programs [14]. Introduction of more compact and user-friendly dialysis machines has also changed the way that patients are dialyzed at home. The aim of this study is to describe patient and treatment factors, short-term biochemical outcomes, and long-term clinical outcomes in HHD patients at European centers that participate in the Knowledge to Improve Home Dialysis Network in Europe (KIHDNEy). The overarching aim of KIHDNEy is to evaluate an array of outcomes on intensive HHD with low-volume dialysate, a newer modality in Europe than in North America.

## Methods

### Study cohort

We performed a retrospective cohort study of anonymized data that were voluntarily provided by 7 dialysis centers throughout Europe. Centers were in England (Portsmouth), France (Caen, Paris), Italy (Bari, Niguarda [Milan]), and Spain (Madrid, Navarre). All patients in the KIHDNEy cohort initiated HHD with the NxStage System One (NxStage Medical, Lawrence, Massachusetts, United States), a portable hemodialysis machine that employs a low volume of lactate-buffered, ultrapure dialysate per session and inverts the traditional ratio between dialysate and blood flow rates. Patients used either a set of 5-l bags of sterile, premixed dialysate (“Express System”) or fluid that was produced in the home with 5-l bags of dialysate concentrate and purified tap water (“PureFlow SL”), without need for a reverse osmosis system. Preceding initiation of HHD, patients were educated in a health care facility about the practice of HHD (including cannulation) and technical aspects of the machine.

### Patient factors

During 2015, centers entered data into a standardized Microsoft Excel worksheet. Data comprised patient characteristics; HHD prescription factors; standard *Kt/V*_urea_ and ultrafiltration volume after 3 and 6 months of HHD; pre-dialysis biochemical parameters at baseline and after 3 and 6 months of HHD; and medication use at baseline and after 3 and 6 months of HHD. Medications comprised erythropoiesis-stimulating agents, heparin, antihypertensive agents, and phosphate binders; units of darbepoetin alfa were converted to units of epoetin alfa-equivalent by multiplying by 250. We also derived ultrafiltration rate after 3 and 6 months of HHD, but with hemodialysis session duration and weight ascertained at baseline. In addition, centers entered data regarding long-term outcomes, including kidney transplant, return to in-center hemodialysis, and death. Patients were followed until October 31, 2015.

### Statistical analysis

Centers were instructed to enter data only for patients who completed ≥6 months of HHD. Therefore, we retained patients who initiated HHD no later than April 30, 2015, and excluded any retained patient with < 6 months of follow-up. Furthermore, we excluded patients with missing data regarding HHD prescription factors. We used descriptive analysis to assess patient characteristics and HHD prescription factors. For each clearance and biochemical parameter, we calculated the mean, standard deviation, median, interquartile range, and 10th–90th percentile interval at baseline (as applicable) and months 3 and 6. For each parameter, we assessed statistical significance of the linear trend across measured times; the test was derived from a linear mixed model of the parameter regressed on time, with random effects (intercept and slope) for each patient, but without further covariate adjustment. With long-term follow-up, we estimated the cumulative incidence of kidney transplant, return to in-center hemodialysis, and death, beginning at 6 months after HHD initiation and ending at the earlier of 48 months after HHD initiation or October 31, 2015. All statistical analyses were performed with SAS, version 9.4 (Cary, North Carolina, United States) and R, version 3.2.3 (Vienna, Austria).

## Results

**Fig. 1:**
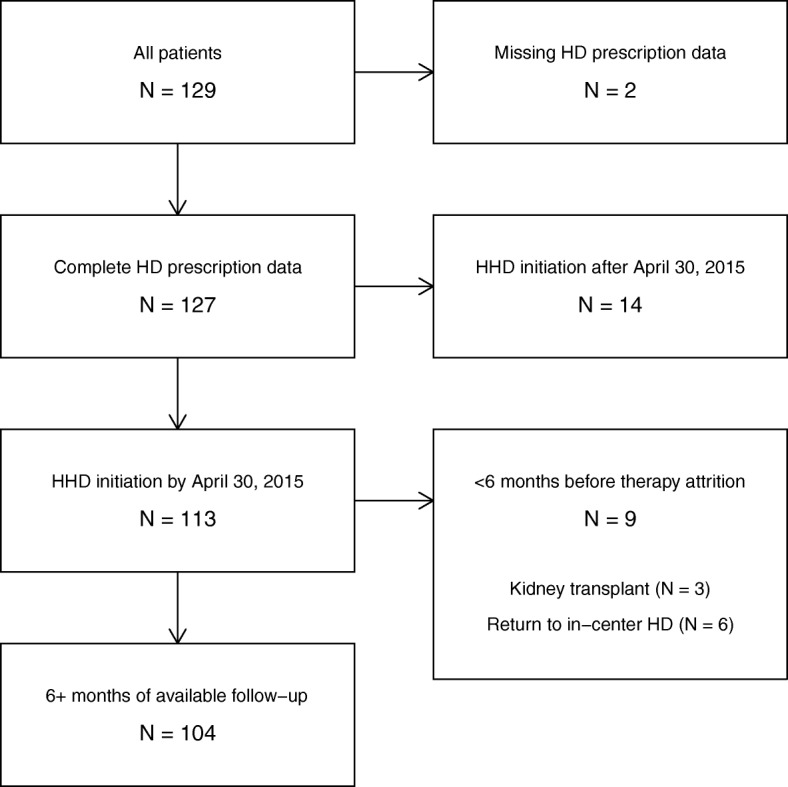
Sample size of study cohort, with iterative application of inclusion criteria. Abbreviations: HD, hemodialysis; HHD, home hemodialysis

We collected data regarding 129 patients in 7 centers. After we excluded patients with missing data regarding the home hemodialysis prescription (*n* = 2), patients who initiated HHD after April 30, 2015 (*n* = 14), and patients with < 6 months of HHD before therapy attrition (*n* = 9), we retained 104 (81%) patients for analysis (Fig. 1). Therapy attrition was attributable to kidney transplant and return to in-center HD; the latter was due to both medical complications and psychosocial issues. Among retained patients, we identified 59 (57%) patients in England, 23 (22%) in France, 10 (10%) in Italy, and 12 (12%) in Spain.

Patient characteristics are displayed in Table 1. Mean age was 49 years (range, 19 to 75 years) and 66% of patients were male. Merely 15% of patients had either diabetes mellitus or hypertensive nephrosclerosis as their primary renal diagnosis. Prevalence of moderate (body mass index, 30–34 kg/m^2^), severe (35–39 kg/m^2^), and morbid (≥40 kg/m^2^) obesity was 13%, 6%, and 3%, respectively. The Charlson comorbidity score was ≤6 points in nearly all patients. Most patients were on conventional hemodialysis before HHD initiation; only 16% of patients were incident cases of end stage renal disease (ESRD).

**Table 1:** Home hemodialysis patient characteristics

	Statistic
Age (years)	
Mean (SD)	49.3 (12.8)
Median (IQR)	49 (19)
10th–90th percentile interval	31.6–65.0
Sex (%)	
Female	33.7
Male	66.3
Primary renal diagnosis (%)	
Diabetes mellitus	9.6
Hypertensive nephrosclerosis	5.8
Glomerulonephritis	29.8
Polycystic kidney disease	10.6
Other diagnosis	44.2
Body mass index (kg/m^2^)	
Mean (SD)	26.7 (6.0)
Median (IQR)	25.7 (7.5)
10th–90th percentile interval	20.4–34.3
Charlson comorbidity score (points)	
Mean (SD)	3.7 (2.0)
Median (IQR)	3 (3)
10th–90th percentile interval	2–6
Prior renal replacement modality (%)	
Conventional hemodialysis	70.2
Intensive hemodialysis	3.8
Peritoneal dialysis	6.7
Kidney transplant	2.9
Incident ESRD	16.3
Prior dialysis duration (months)	
Mean (SD)	36.9 (55.2)
Median (IQR)	18 (35)
10th–90th percentile interval	0–127
History of kidney transplant (%)	
No	63.5
Yes	36.5

Abbreviations: *ESRD* end stage renal disease, *IQR* interquartile range, *SD* standard deviation

**Fig. 2:**
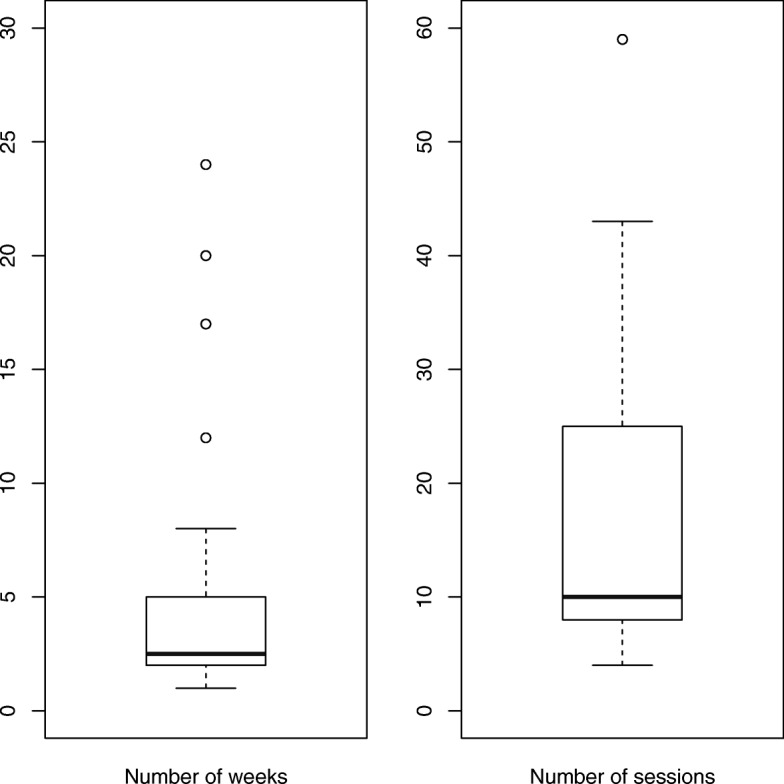
Box plots of numbers of home hemodialysis training weeks and sessions. Each plot displays the median (bold line), the interquartile range (box), and outlying intervals less than or equal to 1.5 times the interquartile range (whiskers)

HHD prescription factors are displayed in Table 2. Over 70% of patients were prescribed 6 sessions per week. Mean (standard deviation) hemodialysis hours per session and per week were 2.6 (0.4) and 15.0 (2.9), respectively, and more than 68% received ≥15 h per week. Dialysate preparation varied by country: in France and Italy, only premixed dialysate was used; in Spain, only dialysate concentrate was used; and in the United Kingdom, both preparations were used, although dialysate concentrate was dominant (86%). Most patients dialyzed with a fistula. Regarding the number of training weeks, mean and median (interquartile range) estimates were 3.8 and 2.5 (3.0), respectively; regarding the number of training sessions, corresponding estimates were 16.8 and 10.0 (16.5) respectively (Fig. 2). Training duration was ≤2 weeks in 50% of all patients and 80% of patients in the United Kingdom.

**Table 2:** Home hemodialysis prescription factors

	Percentage
Hemodialysis sessions per week (%)	
4	1.9
5	24.0
6	70.2
7	3.8
Hours per hemodialysis session (%)	
2.0–2.4	30.8
2.5–2.9	41.3
3.0–3.4	22.1
≥ 3.5	5.8
Hemodialysis hours per week (%)	
10.0–11.9	7.7
12.0–14.9	24.0
15.0–17.9	47.1
≥ 18.0	21.2
Dialysate preparation (%)	
Premixed dialysate	39.4
Dialysate concentrate	60.6
Dialysate liters per session (%)	
15	2.9
20	39.4
25	31.7
30	25.0
40	1.0
Vascular access modality (%)	
Catheter	17.3
Graft	2.9
Fistula	79.8
Cannulation technique ^a^ (%)	
Sharp needle	8.4
Buttonhole needle	80.7
Plastic needle	10.8

^a^ In patients with a fistula

Details of hemodialytic clearance are displayed in Table 3. With HHD, mean standard *Kt/V*_urea_ was nearly 2.7 at months 3 and 6 and roughly 90% of patients had standard *Kt/V*_urea_ ≥ 2.1. Among the minority of patients with recorded ultrafiltration volume at months 3 and 6, mean ultrafiltration volume was roughly 1 L, mean ultrafiltration rate was < 7 mL/hour/kg, and over 80% of patients had ultrafiltration rate < 10 mL/hour/kg.

**Table 3:** Urea clearance, ultrafiltration volume, and ultrafiltration rate in home hemodialysis patients

	Month 3	Month 6
Standard *Kt/V*_urea_		
Patients (*n*)	87	87
Mean (SD)	2.67 (0.49)	2.69 (0.55)
Median (IQR)	2.63 (0.46)	2.63 (0.57)
10th–90th percentile interval	2.14–3.30	2.09–3.49
Ultrafiltration volume ^a^ (L)		
Patients (*n*)	32	32
Mean (SD)	1.07 (0.84)	1.08 (0.87)
Median (IQR)	1.00 (1.02)	1.00 (0.92)
10th–90th percentile interval	0.20–2.00	0.20–2.00
Ultrafiltration rate (mL/hour/kg)		
Patients (*n*)	32	32
Mean (SD)	6.54 (4.85)	6.82 (5.61)
Median (IQR)	6.21 (6.35)	6.46 (5.11)
10th–90th percentile interval	1.51–13.95	0.76–11.88

Abbreviations: *IQR* interquartile range, *SD* standard deviation

^a^ Per hemodialysis session

Biochemical parameters are displayed in Table 4. Mean changes between baseline and months 3 and 6 were modest. Serum bicarbonate increased by 1 mmol/L between baseline and month 6, whereas serum potassium decreased by 0.2 mmol/L. Calcium and phosphorus were stable, while serum albumin and C-reactive protein increased during follow-up. The percentage of patients with serum C-reactive protein > 10 mg/L increased from 22% at baseline to 30% at month 6.

**Table 4:** Biochemical parameters in home hemodialysis patients

	Baseline	Month 3	Month 6	*P* ^a^
Bicarbonate (mmol/L)				
Mean (SD)	23.1 (3.5)	24.1 (2.8)	24.1 (2.9)	0.01
Median (IQR)	23.3 (4.2)	24.0 (4.0)	24.0 (3.5)	
10th–90th percentile interval	18.0–27.3	20.7–27.5	20.0–28.0	
Potassium (mmol/L)				
Mean (SD)	4.80 (0.63)	4.64 (0.71)	4.59 (0.78)	0.01
Median (IQR)	4.80 (0.90)	4.60 (1.10)	4.40 (1.00)	
10th–90th percentile interval	3.9–5.6	3.8–5.5	3.8–5.6	
Calcium (mmol/L)				
Mean (SD)	2.29 (0.19)	2.30 (0.20)	2.28 (0.19)	0.43
Median (IQR)	2.30 (0.24)	2.29 (0.22)	2.30 (0.23)	
10th–90th percentile interval	2.03–2.52	2.06–2.53	2.04–2.49	
Phosphorus (mmol/L)				
Mean (SD)	1.73 (0.49)	1.67 (0.49)	1.68 (0.48)	0.38
Median (IQR)	1.64 (0.72)	1.64 (0.66)	1.62 (0.60)	
10th–90th percentile interval	1.16–2.40	1.10–2.33	1.11–2.28	
Albumin (g/L)				
Mean (SD)	36.8 (5.2)	37.5 (4.0)	37.8 (4.5)	0.03
Median (IQR)	37.0 (5.0)	37.0 (5.0)	38.0 (6.0)	
10th–90th percentile interval	31.0–43.0	32.3–43.0	38.0–50.0	
Hemoglobin (g/dL)				
Mean (SD)	11.4 (1.5)	11.1 (1.6)	11.2 (1.4)	0.25
Median (IQR)	11.5 (1.7)	11.1 (1.9)	11.1 (2.0)	
10th–90th percentile interval	9.2–13.5	9.2–13.0	9.5–13.1	
Beta-2-microglobulin (mg/L)				
Mean (SD)	23.6 (9.7)	26.8 (12.5)	25.4 (10.8)	0.64
Median (IQR)	22.0 (14.7)	25.3 (15.1)	24.0 (14.2)	
10th–90th percentile interval	10.3–36.3	14.4–42.7	13.7–42.0	
C-reactive protein (mg/L)				
Mean (SD)	7.3 (8.9)	11.4 (16.6)	12.4 (24.8)	0.05
Median (IQR)	4.4 (7.2)	5.0 (12.3)	5.0 (12.0)	
10th–90th percentile interval	1.0–18.0	0.7–31.0	0.9–30.0	

Abbreviations: *IQR* interquartile range, *SD* standard deviation

^a^ From test of linear trend across displayed times

Medication use is displayed in Table 5. The mean number of antihypertensive agents per day fell significantly (*P* < 0.001), from 1.46 at baseline to 1.01 at month 6. The percentage of patients using no antihypertensive agents increased from 31% at baseline to 41% at month 6, while the percentage of patients using > 2 antihypertensive agents per day decreased from 23 to 11%. Phosphate binder pill count and ESA dose were stable. The percentage of patients using no heparin for anticoagulation nearly doubled between baseline and month 6 (*P* < 0.001).

**Table 5:** Medication use in home hemodialysis patients

	Baseline	Month 3	Month 6	*P* ^a^
Antihypertensive medication use (agents/day)				
Mean (SD)	1.46 (1.49)	1.10 (1.29)	1.01 (1.11)	< 0.001
Median (IQR)	1 (2)	1 (1)	1 (1)	
10th–90th percentile interval	0–4	0–3	0–3	
Phosphate binder use (pills/day)				
Mean (SD)	3.25 (2.91)	3.13 (2.66)	3.21 (2.84)	0.83
Median (IQR)	3 (4)	3 (3)	3 (5)	
10th–90th percentile interval	0–6	0–6	0–6	
ESA dose (EPO-equivalent IU/week)				
Mean (SD)	8792 (6936)	8211 (6654)	8551 (7086)	0.57
Median (IQR)	8000 (6000)	7750 (6000)	8000 (8000)	
10th–90th percentile interval	2000–17,000	1250–16,000	0–16,125	
Anticoagulant use ^b^ (%)				
No	21.4	NR	40.2	< 0.001
Yes	78.6	NR	59.8	

Abbreviations: *EPO* epoetin alfa, *ESA* erythropoiesis-stimulating agent, *IQR* interquartile range, *IU* international units, *NR* not recorded, *SD* standard deviation

^a^ From test of linear trend across displayed times

^b^ Heparin use during hemodialysis session

**Fig. 3:**
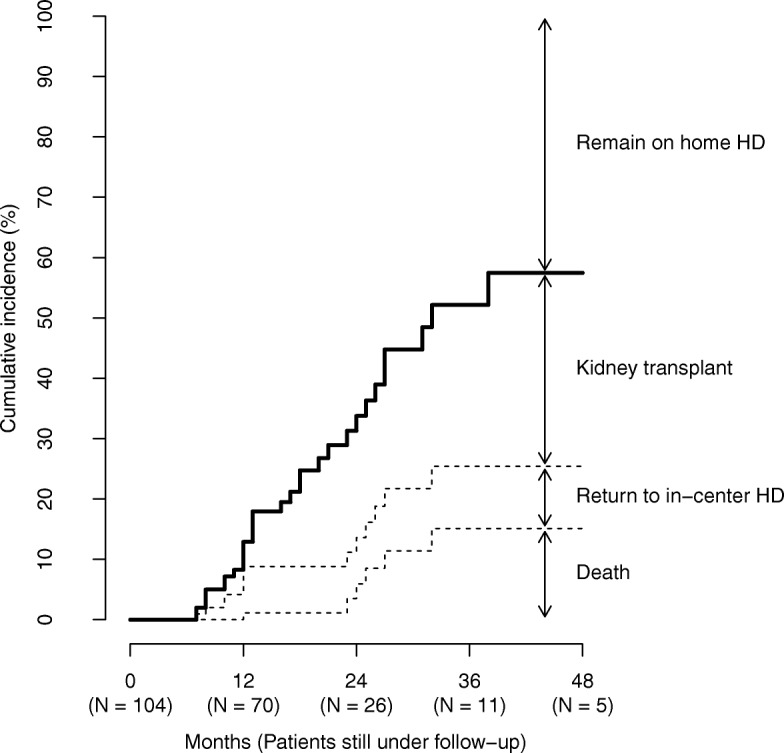
Cumulative incidence of home hemodialysis cessation due to kidney transplant, return to in-center hemodialysis, and death. Abbreviations: HD, hemodialysis

Mean follow-up duration was 18.8 months. During follow-up, there were 17 kidney transplants, 8 returns to in-center hemodialysis, and 6 deaths. After the first 6 months of follow-up, transplant and death rates were 15.3 and 5.4 events per 100 patient-years, respectively. At 24 months after HHD initiation, the cumulative incidence of kidney transplant, return to in-center hemodialysis, and death was 20%, 8%, and 6%, respectively. At 48 months after HHD initiation, corresponding estimates of cumulative incidence were 32%, 10%, and 15%. Thus, approximately 43% of patients remained on HHD at 48 months after HHD initiation (Fig. 3). Returns to in-center hemodialysis were due to medical complications in 2 cases and psychosocial problems in 6 cases.

## Discussion

HHD continues to have limited penetration in Europe and is not currently offered as an option to all ESRD patients. In the most recent annual report of the European Renal Association-European Dialysis and Transplant Association (ERA-EDTA), the highest percentages of dialysis patients on HHD were in Finland (7.1%), Denmark (5.8%), the Netherlands (4.4%), the UK (4.3%), and Sweden (3.6%); corresponding percentages in all other countries were less than 3% [15]. The reasons for low use of HHD are myriad, but primary reasons include the relative simplicity of peritoneal dialysis and the shortage of patient-friendly hemodialysis equipment for application in the home setting [16]. In the KIHDNEy cohort, HHD patients used equipment that employs lactate-buffered, ultrapure dialysate, which is either supplied in premixed bags or produced from concentrate and tap water (without a reverse osmosis system). Each treatment consumes 15 to 60 l of dialysate, which flow at a maximum rate of 200 mL/minute. From the patient’s perspective, the device presents a relatively simple user interface, is sufficiently small to transport, and requires neither electrical nor plumbing modifications to the home; the costs of those modifications are ordinarily between $1000 and $4000 [17]. In this observational study, we evaluated patient and treatment factors, biochemical outcomes, and clinical outcomes in European patients on intensive HHD with low-volume dialysate. Despite its modest sample size, this study is the largest study of European patients on such treatment.

The mean age of patients in the KIHDNEy cohort was almost 50 years. That is roughly 10 years less than the mean age of prevalent ESRD patients in many European countries [15], but similar to the relative youthfulness of HHD patients in the United States [18, 19]. The majority of patients were male, in concordance with HHD populations in North America, Australia, and New Zealand [18, 20, 21]. Furthermore, the prevalence of diabetes as the primary etiology of ESRD was low. However, characterization of the KIHDNEy cohort as a collection of young, healthy men is inaccurate. Age ranged from 19 to 75 years and the Charlson comorbidity score ranged from 2 to 10 points. Notably, body mass index ranged from 13 (underweight) to 51 (obese). Thus, HHD appears to be a viable dialytic modality across a wide array of patients, including patients in poorer health. This observation contradicts the assumption that HHD is feasible exclusively in younger patients with limited comorbidity. In the KIHDNEy cohort, more than 70% of patients converted from in-center hemodialysis to HHD, and mean dialysis duration at HHD initiation was more than 3 years. In the United States, HHD is also commonly prescribed subsequently to in-center hemodialysis [18].

Almost all patients in the KIHDNEy cohort were prescribed either 5 or 6 treatments per week. Because treatment duration was typically between 2.0 and 3.5 h, over 70% of patients accumulated at least 15 treatment hours per week. By comparison, large majorities of in-center hemodialysis patients in France, Italy, Spain, and the UK accumulate no more than 12 treatment hours per week [22]. The ease of delivering more treatment hours per week in the home setting is one of the primary advantages of HHD. The most commonly prescribed dialysate volume was 20 l per session and most patients dialyzed against 20 to 30 l per session. The majority of patients used a fistula and cannulated with a buttonhole needle. Buttonhole cannulation may be associated with increased risks of local and systemic infection, although mupirocin prophylaxis may greatly reduce risk [23, 24]. Slightly more than 17% of patients in the KIHDNEy cohort used a catheter. Catheters are widely used by HHD patients in Canada [21] and do not constitute an absolute contraindication to HHD.

HHD training, which is more complex than peritoneal dialysis training, is an obstacle to the growth of HHD. Needed nursing labor and corresponding costs may be substantial. In the Frequent Hemodialysis Network (FHN) Nocturnal Trial, patients required an average of 27.7 training sessions, which spanned between 11 and 59 days [17]. However, patients in that trial used traditional hemodialysis equipment in the home setting. Patients in the KIHDNEy cohort required an average of 16.8 training sessions. This apparent reduction in training intensity may reflect the simple user interface of the equipment. On the other hand, this reduction may reflect that some patients were familiar with self-cannulation before commencing HHD training. More research about the nature of HHD training is needed. Ultimately, equipment that permits more rapid training can improve the economic feasibility of HHD. Despite the rapid pace of training in the KIHDNEy cohort, we observed good patient retention after the first 6 months of HHD. The rate of death was relatively low, whereas the rate of kidney transplant was relatively high. However, the magnitude of the transplant rate may primarily reflect the age distribution of the KIHDNEy cohort, as nearly all patients were non-elderly; whether HHD directly influences the likelihood of transplant is unknown. We also observed that most returns to in-center hemodialysis occurred in the first 12 months of follow-up and were attributable to psychosocial problems. High incidence of technique failure during the first year of HHD has been observed in the United States [19], and in the FHN Nocturnal Trial, HHD increased perceived caregiver burden [25].

Although intensive HHD with low-flow dialysate is widely used in the United States and is associated with lower risk of death and similar risk of hospitalization, relative to thrice-weekly in-center hemodialysis [5, 6], very little data about solute clearance and biochemistry with this therapeutic approach have been published. Kraus et al. reported that mean standard *Kt/V*_urea_ was nearly 2.3 in patients who were prescribed 6 sessions per week [26]. In the KIHDNEy cohort, mean standard *Kt/V*_urea_ was nearly 2.7 at both 3 and 6 months after HHD initiation. Furthermore, roughly 90% of patients achieved standard *Kt/V*_urea_ of ≥2.1, even without accounting for residual function. Adequate small solute clearance with low-volume dialysate is achievable because the dialysate is highly saturated when the blood flow rate is high and the dialysate flow rate is low. On average, ultrafiltration intensity was low. Multiple studies correlate lower ultrafiltration rate with improved survival and shorter post-dialysis recovery time [27–29]. Changes in biochemistry were generally modest. Serum concentrations of calcium, phosphorus, hemoglobin, and beta-2-microglobulin did not change significantly. The absence of a significant decline in serum phosphorus contrasts with the effect of intensive hemodialysis in the FHN Daily Trial [30]. It is possible that while dialytic clearance of phosphorus increased after HHD initiation [31], dietary intake of phosphorus also increased; to that point, serum albumin increased significantly. Mean serum potassium declined after initiation of HHD, although the 10th–90th percentile range was unchanged. The decline probably reflects the effect of shortening the usual interdialytic interval. Finally, serum C-reactive protein (CRP) increased. Serum CRP > 10 mg/L most often reflects acute or chronic inflammation [32]. Infection may have contributed to the increase, as infection – specifically, vascular access infection – has been reported to be a challenge with HHD [33]. We did not collect data regarding vascular access complications, so the incidence of access infection in the KIHDNEy cohort is unknown. However, serum CRP data suggest that dialysis centers should closely monitor signs of infection on HHD, including tenderness and localized redness at the buttonhole site [34].

Regarding medication use, there were no significant changes in either phosphate binder use or erythropoiesis-stimulating agent dose. There was a significant decline in antihypertensive medication use between baseline and follow-up; the same change was observed in the Following Rehabilitation, Economics and Everyday-Dialysis Outcome Measurements (FREEDOM) Study of short daily HHD [35]. Intensive hemodialysis reduces pre-dialysis systolic blood pressure and the need for antihypertensive medications [11, 36]. We did not collect longitudinal data about blood pressure, but the significant decline in medication use is compatible with a reduction in systolic blood pressure. The percentage of patients who did not require heparin administration nearly doubled after 6 months of HHD. Less need for anticoagulation may have been due to the absence of an air-blood interface in the disposable cartridge in the hemodialysis machine, lower session duration, and possibly, better volume control.

The primary limitations of this study are the small sample size and the brief follow-up interval. Many trends in the KIHDNEy cohort suggest hypotheses that require confirmation in prospective studies with larger sample size and longer follow-up. Nevertheless, this study is one of the largest analyses of biochemistry in patients on intensive HHD with low-flow dialysate. A secondary limitation of this study is the absence of controls, with respect to either hemodialysis setting, frequency, or equipment. Trends in the KIHDNEy cohort cannot be attributed directly to the home setting, intensive hemodialysis, or equipment. In addition, all patients in the KIHDNEy cohort were prescribed diurnal HHD; these data offer no insight into biochemical and clinical outcomes on nocturnal HHD with low-volume dialysate.

## Conclusions

HHD is a unique modality, insofar as it offers the opportunity to individualize treatment and specifically, to increase treatment intensity beyond what is typically feasible in the center setting. For HHD to be attractive to both physicians and patients, HHD equipment must maintain provide good clinical outcomes and be sufficiently easy for patients to use. Preliminary data in the KIHDNEy cohort suggest that transportable equipment that employs low-flow dialysate achieves these objectives when patients are prescribed > 3 sessions per week. However, infection may pose a risk in HHD patients. Ultimately, larger studies of European patients are needed to better understand clinical outcomes associated with this emerging therapeutic approach.

## Abbreviations

CKD: Chronic kidney disease
ERA-EDTA: European Renal Association-European Dialysis and Transplant Association
ESRD: End stage renal disease
FHN: Frequent Hemodialysis Network
FREEDOM Study: Following rehabilitation, economics and everyday-dialysis outcome measurements study
HHD: Home hemodialysis
KIHDNEy: The Knowledge to Improve Home Dialysis Network in Europe
NICE: National Institute of Clinical Excellence
RRT: Renal replacement therapy
